# Fruit Physical Features, Phenolic Compounds Profile and Inhibition Activities of Cranberry Cultivars (*Vaccinium macrocarpon*) Compared to Wild-Grown Cranberry (*Vaccinium oxycoccus*)

**DOI:** 10.1007/s11130-019-00737-7

**Published:** 2019-05-16

**Authors:** Agnieszka Narwojsz, Małgorzata Tańska, Barbara Mazur, Eulalia Julitta Borowska

**Affiliations:** 1 Department of Human Nutrition, Faculty of Food Sciences, University of Warmia and Mazury, ul. Słoneczna 45F, 10-718 Olsztyn, Poland; 2 Department of Plant Raw Materials Processing and Chemistry, Faculty of Food Sciences, University of Warmia and Mazury, Plac Cieszyński 1, 10-726 Olsztyn, Poland

**Keywords:** Geometric features, Colour, Phenolic compounds, Antioxidant activity, Antitrypsin activity, Cranberry fruits

## Abstract

This study analysed fruits of cranberry cultivars: Ben Lear, Bergman, Early Richard, Pilgrim and Stevens and compared them with wild-grown cranberry fruits. The fruits were characterised in terms of dimensions, colour, content of total phenolic compounds, flavonoids, proanthocyanidins and anthocyanins, and hydroxyl radical and trypsin inhibition activities. It was shown that the wild-grown cranberry fruits were characterised by much smaller dimensions and redder colour than fruits of the cranberry cultivars. The most phenolic compounds were found in the Early Richard fruits (357.6 mg/100 g fw), and they showed the highest antitrypsin activity. The highest anthocyanin content (60.6 mg/100 g fw) was determined in the Pilgrim fruits, while the Ben Lear fruits were the richest source of proanthocyanidins (27.9 mg/100 g fw). The antioxidant activity was correlated with the content of phenolic compounds, flavonoids and proanthocyanidins, while the antitrypsin activity was correlated with phenolic compounds and anthocyanin contents.

## Introduction

American cranberry (*Vaccinium macrocarpon*), also known as large cranberry, belongs to the *Ericaceae* family [1]. It is a perennial, evergreen, low-stemmed shrub. Its fruits are spherical red berries [2]. Due to the increasingly rare occurrence of cranberries in the natural state, both the breeding and the cultivation of this species were undertaken. The largest numbers of multi-fruit cranberry plantations are in the United States, Canada, the United Kingdom and the Netherlands, with over 90% of cranberry being produced in the USA and Canada [3, 4]. In recent years, interest in large-fruited cranberry cultivation has also increased in Poland. This is an alternative to wild growing swamp cranberry (*Vaccinium oxycoccus*), which is characterised by smaller fruits compared to large cranberry fruits [3].

Cranberry fruits are a source of bioactive components valuable for the body. They contain vitamins (A, C and E), minerals (potassium, sodium, selenium), as well as lutein and β-carotene [2]. The most important group of health-promoting compounds contained in cranberry fruits are polyphenols, including flavonols, anthocyanins, proanthocyanidins, phenolic acids and resveratrol [4–6]. The content of phenolic compounds depends on the cultivar [2, 3, 7–9], growing season, cultivation conditions [10], maturation [4, 9], time [4, 10] and temperature storage [7]. Cranberry fruits display antioxidant, radical scavenging, anti-inflammatory, antimutagenic, antiviral, antibacterial and antifungal activities [1, 4, 6, 8, 11]. Cranberry has a variety of pro-health action. Fruit juice, tablets or capsules are used to treat urinary tract infections, since the proanthocyanidins contained in the juice prevent the adhesion of the *Escherichia coli* bacteria that cause this infection [1, 11]. It is believed that the “A-type” proanthocyanidins are responsible for this effect [6]. Cranberry ingredients also prevent the adhesion of *Helicobacter pylori* bacteria which cause stomach ulcers [1, 4, 5, 11]. Furthermore, cranberry fruit juice is used in the treatment of periodontitis [12]. It has also been shown that bioactive compounds of cranberry fruits reduce the risk of cardiovascular disease [1, 4, 6, 8].

Due to the growing interest in large-fruited cranberry cultivation in Poland, the characterization of five cultivars was conducted in terms of geometric features, colour, and content of total phenolic compounds, flavonoids, proanthocyanidins, anthocyanins. These physical and chemical properties were correlated with antioxidant activity and also antitrypsin activity, which has not been previously studied in the cranberry fruits. Additionally, the anthocyanin content in fruit peels was also determined. The reference sample consisted of wild-grown cranberry fruits.

## Materials and Methods

### Plant Material

The research material comprised the fruits of five cultivars of large cranberry: Ben Lear, Bergman, Early Richard, Pilgrim and Stevens. The fruits came from the Experimental Field of Blueberries in the Department of Horticulture and Natural Basics of Horticulture at SGGW in the village of Błonie near Piaseczno. The fruit harvest was carried out in 2016 after berries were ripe for consumption. The fruits of wild-grown cranberry collected from the natural environment near Olsztyn were then compared.

### Physical Analysis

The geometrical features (diameter, length, width and circularity) and surface colour (expressed in CIEL*a*b* model, where L* - lightness, a* - greenness/redness, b* - blueness/yellowness) of cranberries were measured using digital image analysis (DIA) according to the method described. The images were acquired by a high resolution, low-noise CCD (charge-coupled device) Nikon DXM-1200 colour camera (Nikon Inc., Melville, USA) and analysed by LUCIA v. 4.8 software (Laboratory Imaging, Prague, Czech Republic). The frame grabber was at a resolution of 1280 × 1024 pixels. The light source was a Kaiser RB 5004 HF – High Frequency Daylight Copy Light set with 4 × 36 W fluorescent light tubes (colour temperature about 5400 K) (Kaiser Fototechnik GmbH & Co.KG, Buchen, Germany). Before analyses, the calibration to a standard white reflective plate was done.

### Phenolic Compounds Analyses

Methanol (80% solution containing 0.1% hydrochloric acid) was used to prepare phenolic extracts based on the procedure reported by Borowska et al. [3]. The content of total phenolic compounds was determined using the Folin-Ciocalteu reagent according to the procedure described by Borowska et al. [3]. The results were expressed as milligrams of gallic acid equivalent (GAE) *per *100 g of fresh weight (fw). The total flavonoid content was determined according to the method described by Lamaison and Carnat [13]. The results were expressed as milligrams of quercetin equivalent (QE) *per* 100 g fw. The total anthocyanin content in fruits and peels was determined using the pH differential method according to the method given by Borowska et al. [3]. To determine the content of anthocyanins in fruit peels, the fruit peel was separated by hand. The preparation of the extract from peels was similar to that of whole cranberry fruits. The anthocyanins content was expressed as milligrams of cyanidin-3-glucoside equivalent (C3GE) *per* 100 g fw. Determination of the total content of proanthocyanidins was carried out according to the procedure using 1-butanol-HCl reagent described by Mole and Waterman [14]. The proanthocyanidin content was expressed as milligrams of catechin equivalent (CAE) *per* 100 g fw. The anthocyanin profile in fruits was determined according to the procedure of Borowska et al. [3]. The anthocyanins were extracted with 10% aqueous formic acid solution. A HPLC system consisted of a Hewlett-Packard 1050 (Hewlett-Packard, Palo-Alto, CA, USA) liquid chromatograph equipped with a diode array detector. A Li Chrospher C18 column (250 × 4.6 mm) with a particle size of 5 μm was used for the assay. The injection volume was 10 μl. The mobile phase was a mixture of water: acetonitrile: formic acid (81:9:10, *v*/v/v). The flow rate was 1.1 ml/min and the temperature of the oven column was 30 °C. The wavelength was set to 520 nm. The identification was made by comparing peak retention times of the samples with anthocyanin standards and literature data. The amount of anthocyanins was calculated from the calibration curves. The calibration curve was plotted for each standard. The concentration of the standard ranged from 10 to 100.03 mg/l. The correlation of the calibration curve was r^2^ = 0.9899. The content of individual anthocyanins was expressed in milligrams *per* 100 g fw.

### Inhibition Activities Analyses

A scavenging of the hydroxyl radical (OH) was measured by the deoxyribose method [3]. The results were expressed as μmol of Trolox equivalent (TE) *per* g fw. The antitrypsin activity was determined according to the method given by Guillamón et al. [15]. The activity of trypsin inhibitors was expressed in TUI units converted to 1 mg fw.

### Statistical Analysis

The obtained data were analysed statistically using Statistica 12.0 PL software (StatSoft, Kraków, Poland). A one-way analysis of variance (ANOVA) with the Tukey test was used. Pearson’s correlation coefficients were also determined to establish the relationship between phenolics and colour as well as antioxidant and antitrypsin activities. The statistical significance was set at the 5% level.

## Results and Discussion

**Fig. 1:**
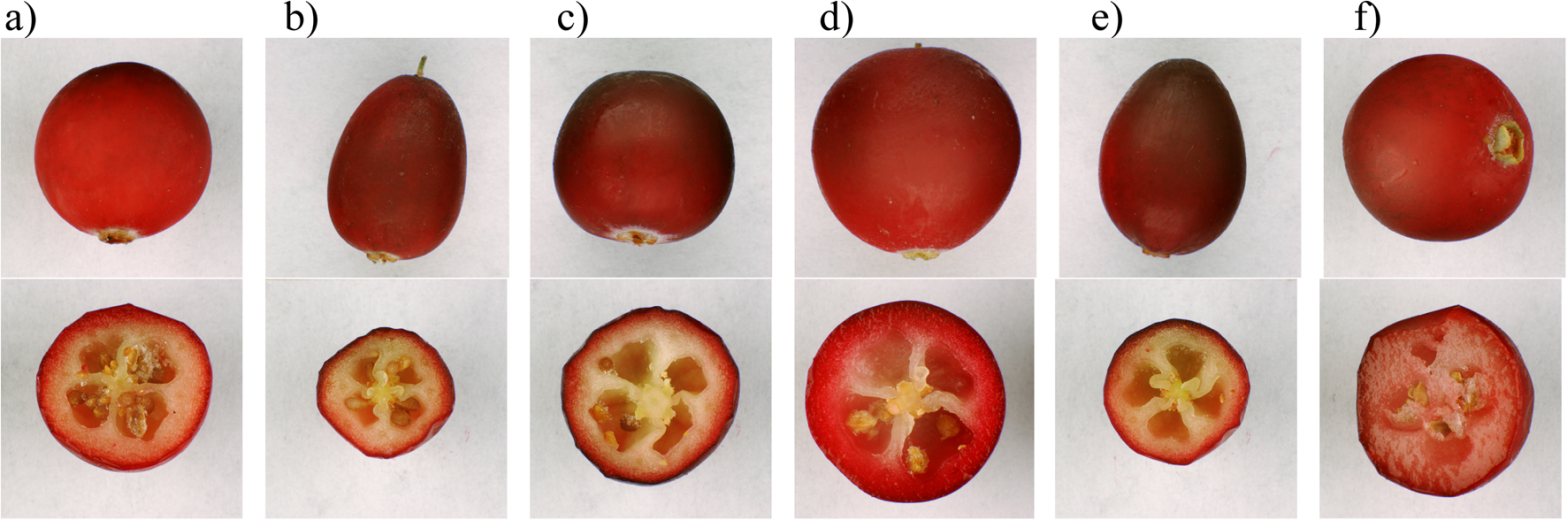
Images of surfaces and cross-sections of the fruits of cranberry cultivars: Bean Lear (**a**), Bergman (**b**), Early Richard (**c**), Pilgrim (**d**), Stevens (**e**), and wild-grown cranberry (**f**)

The fruits of the cranberry cultivars were significantly different (*P* ≤ 0.05) in terms of their dimensions. The average diameter values ranged from 12.35 mm for fruits of the Ben Lear cultivar to 17.20 mm for the fruit of the Pilgrim cultivar. The average width and length values for fruits of cranberry cultivars were 11.95–15.60 mm and 12.99–19.04 mm, respectively (Table 1). The wild-grown cranberry fruits were characterised by much smaller dimensions. For example, the diameter of these fruits was 13.6–38.0% smaller than the diameter of fruits of cranberry cultivars. However, the wild-grown cranberry fruits were more uniform in size (CV in the range 4.0–6.5%) than fruits of the cranberry cultivars (CV in the range of 10.1–14.2%). Generally, the least aligned fruits were in the Pilgrim cultivar. The fruits of this cultivar were also distinguished by its size, which is also visible in Fig. 1. The shape of cranberry fruits was not very diverse. The fruits of Stevens and Bergman cultivars were characterised by only slightly smaller aspect ratios - circularity (0.92–0.93) than fruits of other cranberry cultivars (0.95) and wild-grown cranberry (0.95). Circularity turned out to be the least diversified geometric feature of fruits within the studied cultivars (CV <5%).

**Table 1:** The physical features of the studied cranberry fruits

Feature	Cranberry cultivars					Wild-grown cranberry
	Ben Lear	Bergman	Early Richard	Pilgrim	Stevens	
Geometrical features						
Diameter (mm)	12.35 ± 1.30^a^	14.04 ± 1.59^b^	15.16 ± 1.60^d^	17.20 ± 1.97^e^	14.65 ± 1.49^c^	10.67 ± 0.43^a^
*CV (%)*	10.5	11.4	10.6	11.5	10.2	4.0
Length (mm)	12.99 ± 1.49^a^	15.89 ± 1.65^b^	16.16 ± 1.69^b^	19.04 ± 2.41^d^	16.96 ± 1.87^c^	11.46 ± 0.75^a^
*CV (%)*	11.5	10.4	10.5	12.7	11.1	6.5
Width (mm)	11.95 ± 1.21^ab^	12.67 ± 1.80^b^	14.52 ± 1.61^d^	15.60 ± 1.84^e^	12.96 ± 1.35^c^	9.69 ± 0.48^a^
*CV (%)*	10.1	14.2	11.1	11.8	10.4	5.0
Circularity (−)	0.95 ± 0.03^b^	0.93 ± 0.03^a^	0.95 ± 0.03^b^	0.95 ± 0.03^b^	0.92 ± 0.04^a^	0.95 ± 0.01^b^
*CV (%)*	2.9	3.2	3.1	2.9	4.3	1.1
Colour parameters						
L* (%)	60.70 ± 4.48^d^	60.13 ± 6.07^c^	56.14 ± 6.94^a^	59.19 ± 7.15^c^	58.02 ± 4.77^b^	59.28 ± 1.95^c^
*CV (%)*	7.4	10.1	12.4	12.0	8.2	3.3
a* (−)	27.37 ± 5.36^a^	31.68 ± 6.50^b^	27.95 ± 5.73^a^	34.34 ± 7.34^c^	30.76 ± 6.00^b^	35.76 ± 1.97^d^
*CV (%)*	19.6	20.5	20.5	21.4	19.5	5.5
b* (−)	24.92 ± 3.34^b^	27.21 ± 3.41^d^	24.99 ± 3.11^b^	28.18 ± 4.40^e^	26.19 ± 3.07^c^	20.37 ± 2.31^a^
*CV (%)*	13.4	12.5	12.4	15.6	11.7	11.3

Data are expressed as mean value ± standard deviation; CV – variation coefficient; *n* = 500

Values in the same line with different superscript letters are significantly different (*P* ≤ 0.05)

The fruit colour of the tested cultivars was differentiated (Table 1). The L* component, indicating the lightness of the colour, assumed average values in the range of 56.14–60.70%. The fruits of the Ben Lear cultivar were characterised by the brightest colour, and the Early Richard fruits were the darkest. This is also confirmed by photographs of fruits (Fig. 1). In terms of redness, which was indicated by the values of the component a*, the fruits of wild-grown cranberry were the most distinguished. The average value of the a* component for these fruits was 35.76 and was higher than for fruits of the cranberry cultivars by 4.10–30.60%. The least red were the fruits of the Ben Lear cultivar (a* = 27.37). The b* component also allowed the wild-grown cranberry fruits to be distinguished from the fruits of cranberry cultivars. However, it received the lowest values for the wild-grown cranberry (b* = 20.37). The most yellow in colour (b* = 28.18) was found in the Pilgrim cultivar fruits. On the other hand, the Pilgrim fruits were the most heterogeneous in terms of peel colour, as indicated by the highest variability coefficient values (CV up to 21.4% for a* component). Česonienė et al. [16] studied fruits of 18 clones of wild-grown cranberry growing in Lithuania. They show that the shape of wild-grown cranberry fruits had high variability. The most common were clones with round or oblate berries. The average width and height of the fruits varied from 10.0 to 13.5 mm and from 9.9 to 14.1 mm, respectively. The colour of the fruits was red or dark red at full ripeness. Only two clones with pink fruits were singled out along with one clone with purple fruits. Ruse and Rakcejeva [10] compared Latvian wild and large-berry cranberries. Some of the large-berry cranberries were the same cultivar as in our study. However, we obtained higher values of L* and a* components than in cited work. The L* value for wild-grown cranberry fruits harvested in Poland were similar to these obtained for fruits of Ben Lear, Bergman, Pilgrim and Stevens cultivars. In turn, the cultivars of cranberry harvested in Latvia characterised by a visibly darker fruit colour than wild-grown cranberry. For the a* component, our results show opposite relationships. The cranberry fruits of Pilgrim cultivar harvested in Latvia had the highest intensity of red colour, while fruits of wild cranberry had the lowest intensity, and cranberry fruits of Polish wild cranberry were the reddest.

The total content of phenolic compounds in the fruits of large-fruited cranberry cultivars ranged from 163.4 mg/100 g fw in the Pilgrim cultivar to 357.6 mg/100 g fw in the Early Richard cultivar (Table 2). The variety of cranberry cultivars in terms of the content of phenolic compounds was also studied by Wang and Stretch [7], Borowska et al. [3], Viskelis et al. [9] and Teleszko [2]. The content of polyphenols in the Ben Lear cranberry fruits tested in the current study was 315.9 mg/100 g fw and was consistent with the amount determined in fruits of the same cultivar studied by Zheng and Wang [17]. Viskelis et al. [9] noted a similar amount of polyphenols in the Pilgrim cultivar, while in the Stevens and Ben Lear cultivars they determined smaller amounts of these compounds than in the current study.

**Table 2:** The content of phenolics compounds in the studied cranberry fruits

Phenolic compounds	Cranberry cultivars					Wild-grown cranberry
	Ben Lear	Bergman	Early Richard	Pilgrim	Stevens	
Total polyphenols (mg GAE/100 g fw)	315.9 ± 3.5^e^	261.4 ± 2.2^d^	357.6 ± 3.8^f^	163.4 ± 1.9^a^	222.1 ± 2.4^b^	226.5 ± 2.7^c^
Total flavonoids (mg QE/100 g fw)	114.2 ± 0.7^a^	165.6 ± 0.9^f^	131.5 ± 1.0^b^	138.6 ± 0.5^d^	142.1 ± 0.8^e^	136.0 ± 0.6^c^
Total proanthocyanidin (mg CAE/100 g fw)	27.9 ± 1.4^d^	15.1 ± 1.3^b^	14.3 ± 0.8^b^	14.2 ± 0.7^b^	8.3 ± 0.5^a^	22.4 ± 1.2^c^
Anthocyanins (mg C3GE/100 g fw)						
Total in fruits	44.6 ± 0.9^a^	47.9 ± 1.2^b^	52.2 ± 0.8^c^	60.6 ± 1.3^f^	54.0 ± 1.1^d^	59.1 ± 0.7^e^
Total in peels	153.2 ± 1.7^d^	270.3 ± 2.8^f^	260.2 ± 2.4^e^	114.4 ± 1.9^a^	139.6 ± 2.5^c^	136.1 ± 1.3^b^
Cyanidin-3-galactoside	13.2 ± 0.1^c^	24.0 ± 0.03^d^	13.2 ± 0.2^c^	3.85 ± 0.02^b^	2.86 ± 0.01^a^	3.81 ± 0.02^b^
Cyanidin-3-glucoside	1.29 ± 0.03^a^	1.32 ± 0.02^a^	8.80 ± 0.04^c^	2.56 ± 0.02^b^	19.1 ± 0.1^d^	2.56 ± 0.01^b^
Cyanidin-3-arabinoside	4.65 ± 0.04^c^	3.98 ± 0.02^b^	2.57 ± 0.01^a^	6.85 ± 0.04^e^	6.33 ± 0.02^d^	6.29 ± 0.03^d^
Peonidin-3-galactoside	22.1 ± 0.2^d^	15.1 ± 0.2^a^	18.3 ± 0.1^b^	44.5 ± 0.0^e^	21.3 ± 0.1^c^	43.6 ± 0.1^e^
Peonidin-3-glucoside	1.51 ± 0.01^b^	1.16 ± 0.01^a^	7.67 ± 0.04^f^	2.48 ± 0.03^d^	4.09 ± 0.08^e^	1.95 ± 0.01^c^
Not identified	1.83 ± 0.01^e^	2.35 ± 0.02^f^	1.60 ± 0.01^d^	0.38 ± 0.01^b^	0.30 ± 0.01^a^	0.87 ± 0.02^c^

Data are expressed as mean value ± standard deviation; *n* = 3

Values in the same line with different superscript letters are significantly different (*P* ≤ 0.05)

Smaller differences between the cranberry cultivars were found in the flavonoid content (Table 2). The Bergman cultivar fruits had the highest concentration of these compounds (165.6 mg/100 g fw). The flavonoid contents in fruits of the Early Richard, Pilgrim and Stevens cultivars were similar and amounted to 131.5, 138.6 and 142.1 mg *per* 100 g fw, respectively. The lowest amount of flavonoids was determined for the Ben Lear cultivar (114.2 mg/100 g fw). The content of flavonoids in wild-grown cranberry was 136.0 mg in 100 g of fruits.

As can be seen from the data presented in Table 2, the highest amount of proanthocyanidins, 27.9 mg/100 g fw, was distinguished by the fruits of the Ben Lear cultivar. A large amount of these compounds also contained the fruits of wild-grown cranberry (22.4 mg/100 g fw). The fruits of the Stevens cultivar were definitely the least rich in this group of compounds (8.3 mg/100 g fw). Differences in the content of proanthocyanidins between cultivars were also found in Carpenter et al. [18]. The amount of proanthocyanidins in the fruits of the cultivars tested by them ranged from 18 to 92 mg/g of dry weight.

The studied cranberry fruits contained from 44.6 to 60.6 mg anthocyanins in 100 g fw (Table 2). The fruits of the Pilgrim cultivar proved to be the richest source of these compounds. The Ben Lear cultivar had the lowest amount of anthocyanins. In fruits of the same cultivar studied by Zheng and Wang [17], a smaller amount of anthocyanins (32 mg/100 g fw) was determined. The fruits of the Pilgrim cultivar analysed in the study of Viskelis et al. [9] contained a smaller amount of anthocyanins, compared to the same cultivar studied in this work, although the Ben Lear cultivar had more and the Stevens cultivar was similar. Differences in the content of anthocyanins between cranberry cultivars were noted by Wang and Stretch [7], Borowska et al. [3], Viskelis et al. [9], Teleszko [2], and Ruse and Rakcejeva [10]. A relatively high content of anthocyanins (59.1 mg/100 g fw) was characterised by wild-grown cranberry (Table 2). Ruse and Rakcejeva [10] reported that the fruits of the five large-fruited cranberry cultivars (Steven, Bergman, Ben Lear, Pilgrim and Early Black) contained more anthocyanins than the fruits of wild-grown cranberry fruits. Pappas and Schaich [8] noted that anthocyanins in cranberry fruits are located mainly in the peels. Therefore, in our study the content of these compounds in fruit peels was determined. As can be seen from the data included in Table 2, the studied fruits of particular cranberry cultivars were characterised by a large diversity in terms of the amount of anthocyanins in the peels. The content of these compounds in 100 g fw of fruit peels ranged from 114.4 (Pilgrim cultivar) to 270.3 mg (Bergman cultivar). In turn, 100 g fw of fruit peels of wild-grown cranberry contained 136.1 mg of anthocyanins.

Using the HPLC technique, five anthocyanins were identified in the studied cranberry fruits: cyanidin-3-galactoside, cyanidin-3-glucoside, cyanidin-3-arabinoside, peonidin-3-galactoside and peonidin-3-glucoside (Table 2). In the anthocyanin chromatograms of wild-grown cranberry and cranberry cultivars, the presence of two peaks that were not identified was also shown. Peonidin-3-galactoside proved to be the dominant anthocyanin in all cranberry samples. Its amount ranged from 15.1 mg/100 g fw (Bergman cultivar) to 44.5 mg/100 g fw (Pilgrim cultivar) (Table 2). Zheng and Wang [17] and Viskelis et al. [9] identified six anthocyanins in cranberry fruits: cyanidin-3-galactoside, cyanidin-3-glucoside, cyanidin-3-arabinoside, peonidin-3-galactoside, peonidin-3-glucoside and also peonidin-3-arabinoside, which was not found in the fruits analysed in this work. In the cited work peonidin-3-galactoside dominated, as in our study. Variations in the content of individual anthocyanins determined by the HPLC technique were found by Teleszko [2].

The fruits of studied cranberry cultivars were characterised in terms of scavenging hydroxyl radicals, and the results are shown in Table 3. The highest of OH radical inhibition was characterised by fruits of the Ben Lear cultivar (0.83 μmol TE/g fw) and the Early Richard cultivar (0.81 μmol TE/g fw). The least active (0.65 μmol TE/g fw) were fruits of the Stevens cultivar. Wang and Stretch [7] as well as Oszmiański et al. [19] also documented that the antioxidant activity of cranberry fruits is affected by cultivar. However, Oszmiański et al. [19] found that the Stevens cultivar had both significantly higher concentrations of bioactive compounds and antioxidant capacity in comparison to the Pilgrim and Ben Lear cultivars.

**Table 3:** Antioxidant and antitrypsin activities of the studied cranberry fruits

Activity	Cranberry cultivars					Wild-grown cranberry
	Ben Lear	Bergman	Early Richard	Pilgrim	Stevens	
Antioxidant activity (μmol TE/g fw)	0.83 ± 0.05^b^	0.71 ± 0.07^ab^	0.81 ± 0.04^ab^	0.69 ± 0.09^ab^	0.65 ± 0.03^a^	0.76 ± 0.06^ab^
Antitrypsin activity (TUI/mg fw)	1.93 ± 0.04^d^	1.77 ± 0.06^c^	2.06 ± 0.05^e^	1.15 ± 0.03^a^	1.58 ± 0.03^b^	1.50 ± 0.06^b^

Data are expressed as mean value ± standard deviation; *n* = 3

Values in the same line with different superscript letters are significantly different (*P* ≤ 0.05)

Although low molecular weight protein fractions are responsible for antitrypsin activity, phenolic compounds may also have such properties [20]. While the inhibitory effects of phenolics on the digestion of energy-rich food components (saccharides and lipids) may be regarded as beneficial, primarily in weight-control diets, their inhibitory effects on the digestion of proteins are not desirable due to reduced utilization of amino acids [20]. The evaluated fruits of different cranberry cultivars were characterised by a large variation in antitrypsin activity (Table 3). The highest activity of trypsin inhibition (2.06 TUI/mg fw) was observed in the Early Richard cultivar, followed by the Ben Lear (1.93 TUI/mg fw) and the Bergman cultivars (1.77 TUI/mg fw). Similar trypsin inhibition levels were found for both the Stevens cultivar and the wild-grown cranberry fruits, for which the activity values were 1.58 and 1.50 TUI/mg fw, respectively. The lowest antitrypsin activity (1.15 TUI/mg fw) was found in the Pilgrim cultivar fruits. It should be emphasized that the determined antitrypsin activity of cranberry fruits is several times lower than the leguminous seeds that had by the highest activity among fruits and vegetables [15].

The correlations between selected features of the cranberry fruits are presented in Table 4. The L* value was positively correlated with total proanthocyanidin content, and negatively with cyanidin-3-glucoside and peonidin-3-glucoside. The a* value was negatively correlated with total phenolic compound content with a statistically significant coefficient (r = −0.82). The relationships between a* value and content of total anthocyanins in fruits and two anthocyanins (cyanidin-3-arabinoside and peonidin-3-galactoside) were also observed. For the b* value, an inverse relationship with proanthocyanidin content was noted. Ruse and Rakcejeva [10] also confirmed a close interaction between the a* value and anthocyanin content in cranberry fruits with a coefficient of 0.92. It was shown that the antioxidant activity was dependent on total phenolic, flavonoid and proanthocyanidin contents, but only one anthocyanin (cyanidin-3-arabinoside) influenced this activity. In turn, tripsin inhibitor activity was very strongly and positively correlated with content of total phenolic compounds (r = 0.98). Furthermore, this activity was dependent of anthocyanin content, especially cyanidin-3-arabinoside (r = −0.89). The linear relationship between antioxidant capacity and anthocyanin content was confirmed by Prior et al. [21]. Kalt et al. [22] documented that the antioxidant activity of small fruits (berries) is strongly correlated with the content of both total phenolics (r = 0.83) and total anthocyanins (r = 0.90).

**Table 4:** Correlation coefficients for relationships between phenolic compound content, colour and activities of the studied cranberry fruits

	L*	a*	b*	Antioxidant activity	Antitrypsin activity
Total phenolic compounds	–	−0.82*	–	0.79	0.98*
Flavonoids	–	0.42	–	−0.66	–
Proanthocyanidin	0.59	–	−0.52	0.77	–
Total anthocyanin in fruits	–	0.80	–	−0.47	−0.80
Total anthocyanin in peels	–	−0.44	–	–	0.70
Cyanidin-3-galactoside	–	−0.43	–	–	0.62
Cyanidin-3-glucoside	−0.62	–	–	−0.47	–
Cyanidin-3-arabinoside	–	0.71	–	−0.61	−0.89*
Peonidin-3-galactoside	–	0.79	–	–	−0.81
Peonidin-3-glucoside	−0.97*	−0.42	–	–	0.40

*- correlation coefficient statistically significant at *P ≤ 0.05*

## Conclusions

This study found large variation in the physical properties, content of phenolic compounds as well as antioxidant and antitrypsin activities of cranberry fruits. The fruits of cranberry cultivars were characterised by much higher dimensions than fruits of the wild-grown cranberry. Furthermore, the fruits of wild-grown cranberry had the most red colour and, at the same time, the least yellow colour. In turn, the fruits of the Ben Lear cultivar were characterised by the lightness colour, and the fruits of Early Richard cultivar were the darkest. The Early Richard cranberry sample was also the most heterogeneous in terms of fruit peel colour, while the wild-grown cranberry sample was the most homogeneous. The richest source of polyphenols was the Early Richard cranberry. The fruits of this cultivar were also distinguished by the strongest antitrypsin activity. The fruits of the Bergman cultivar contained the most flavonoids, while the Ben Lear cultivar had the least. The Ben Lear cranberry fruits were distinguished by the highest concentration of proanthocyanidins, while the fruits of the Stevens cultivar contained more than three times less of these compounds. The highest concentration of anthocyanins was determined in the Pilgrim cultivar, while the highest levels of these compounds were found in peels of the Bergman cultivar. The use of the HPLC technique allowed differences in the quantitative composition of individual anthocyanins in the fruits to be shown and, regardless of the cultivar, peonidin-3-galactoside predominated in the fruits. Based on the determined correlation relationships, it was noted that the redder fruits contained more anthocyanins, but fewer other phenolic compounds, which was responsible for their lower antioxidant and antitrypsin activities.
